# Gesture and Sign: Cataclysmic Break or Dynamic Relations?

**DOI:** 10.3389/fpsyg.2018.01651

**Published:** 2018-09-10

**Authors:** Cornelia Müller

**Affiliations:** Faculty of Social and Cultural Studies, European University Viadrina, Frankfurt (Oder), Germany

**Keywords:** gesture and sign, McNeill’s gesture-sign continua, multimodality of language use, singular gestures, recurrent gestures, silent gestures, emblems, conventionalization processes

## Abstract

The goal of the article is to offer a framework against which relations between gesture and sign can be systematically explored beyond the current literature. It does so by (a) reconstructing the history of the discussion in the field of gesture studies, focusing on three leading positions (Kendon, McNeill, and Goldin-Meadow); and (b) by formulating a position to illustrate how this can be achieved. The paper concludes by emphasizing the need for systematic cross-linguistic research on multimodal use of language in its signed and spoken forms.

## Introduction

Throughout the relatively short history of gesture studies, the relation between gesture and sign continues to figure as a central topic. For sign language studies, the question has politically been a highly delicate one, and it remains a vital issue in contemporary sign language research. Fortunately, today, we are in a position to discuss the relation between gesture and sign against the solid background of sign language studies, leaving no doubts concerning the fundamentally linguistic nature of signed languages (Kendon, 2004, 2008, 2014; Steinbach et al., 2012; Goldin-Meadow and Brentari, 2017). Against this background, discussions of the relation between gesture and sign can be very openly reconsidered.

Recent contributions to this discussion are the paper by Susan Goldin-Meadow and Diane Brentari “Gesture, sign, and language: The coming of age of sign language and gesture studies,” published in 2017, and Kendon’s (2014) “Semiotic diversity in utterance production and the ‘concept’ of language.” The two publications come to very different conclusions concerning the relation between gesture and sign. While Kendon’s work on gesture and sign lays out a multitude of ways in which gestures and sign “are on common ground” (Kendon, 2004, chapter 15), Goldin-Meadow highlights differences between gesture and sign early on, postulating a ‘cataclysmic break’ between the two (Singleton et al., 1995). Informed by McNeill’s theory of gesture, this idea involves a focus on spontaneously created gestures and on gestures that “predict learning” (Goldin-Meadow and Brentari, 2017, p. 1).

Kendon, on the other hand, used the term ‘gesture’ in a much broader sense. In his 2004 book, *Gesture: Visible Action as Utterance*, he suggests to use the term ‘gesture’ as “a label for actions that have the features of manifest deliberate expressiveness” (Kendon, 2004, p. 15; see Müller, 2014a for a minute appreciation of Kendon’s notion of gestures as movements displaying deliberate expressiveness). In 2013 he suggests employing “utterance visible action” to refer to what is commonly referred to as ‘gesture’: “In this essay, I offer a survey of the main questions with which I have been engaged in regard to “gesture,” or, as I prefer to call it, and as will be explained below, “utterance visible action.” (Kendon, 2013, p. 7). His work on ‘utterance visible action’ concentrates on hand movements, as indicated in an article the following year: “And although visible bodily actions in the torso, head and face can and do play roles in what is said in an utterance, here I shall concentrate upon the way hand actions interact with what is spoken in the production of content.” (Kendon, 2014, p. 4). Kendon comes to the conclusion that the question of how gesture and sign relate must be shifted to “how visible bodily action is used in utterance construction” and “becomes as much a part of the study of speakers as, necessarily, it is already a part of the study of signers” (Kendon, 2014, p. 13).

Rather than starting with the current positions in that debate, this article offers a historical reconstruction of the discussion of the gesture-sign relation carried out in the field of gesture studies. Why it is useful to look back in close detail? We tend to assume, particularly in psychology and the cognitive sciences, that academic knowledge advances continuously, making publications quickly look ‘outdated’ and ‘overtaken’ by more recent ones. The underlying assumption is that more recent publications are the more knowledgeable and offer the most up to date state of the art of academic research. Sometimes, however, the most recent debates carry the burden of ‘older’ discussions – often implicitly. For the discussion concerning the relation between gesture and sign this is definitely the case. One goal of this paper is therefore to exemplify that a close reading of the history of a scholarly discussion may not only help evaluate current positions, but indeed may still offer valuable insights, ideas, concepts or analytical criteria to work with. Given the scope of this paper, the focus is on the discussion of the relation between gesture and sign as it was led in the field of gesture studies.

Note that what is presented here is a close reading of the writings of Kendon, McNeill, and Goldin-Meadow (and also her 2017 co-author Diane Brentari). Put differently, it is an analysis of their lines or argumentation as presented in the texts. It is not a reconstruction of their current opinions. It aims instead at presenting a history of the development of an academic discussion on the relation between gesture and sign from a point of view of linguistic gesture studies (Müller et al., 2013b). The paper thus presents the author’s view of the arguments. To substantiate this reconstruction, many quotations of the original formulations are included. The figures in the article are analyses of argumentations as reconstructed by the author.

The paper begins with a reconstruction of the relation between gesture and sign in three seminal strands of work: Kendon on gestures, visible actions as utterances, which include sign; McNeill and his reading of Kendon’s work, and his highly influential model of gesture-sign continua; and Goldin-Meadow’s idea of a cataclysmic break between gesture and sign. I show how Kendon’s work highlights commonalities, how McNeill underlines differences and discontinuities, and the grounds on which Goldin-Meadow comes to postulate a categorical divide between gesture and sign.

In the second part of the article, I draw on and develop Kendon’s work to counter Goldin-Meadow’s position. The theoretical framework against which this counter position is formulated adopts a concept of language as inherently multimodal, usage-based, and dynamic (Müller, 2007a, 2008; Müller et al., 2013a) and assumes an understanding of gesture as deliberate expressive movement (Müller, 2014a). The term gesture covers the full spectrum of co-speech gestures: singular, recurrent, and emblematic (Müller, 2010, 2017). The three types of gestures differ with regard to forms and degrees of conventionalization and with regard to their typical linguistic and communicative functions. Singular gestures are created on the spot; although they are based on a culturally shared repertoire of techniques of gesture creation (e.g., gestural modes of representation Müller, 1998a,b, 2014b, 2016), the specific realizations in a given context are rather free and spontaneous. Recurrent gestures “merge conventional and idiosyncratic elements and occupy a place between spontaneously created singular and emblems as fully conventionalized gestural expressions on a continuum of increasing conventionalization,” and “involve emergent de-compositions of gestural movements into smaller concomitant gestalts” (Müller, 2017, p. 276). Emblematic gestures are fully conventionalized gestural movements. Functionally, the three types of gestures differ in that singular gestures mostly serve ‘lexical’ functions, for instance as attributes (Fricke, 2013); recurrent gestures mostly serve pragmatic functions (Bressem and Müller, 2014a; Ladewig, 2014b), as do emblematic gestures (Teßendorf, 2013). However, while singular and recurrent gestures operate upon spoken language utterances (contributing semantically or meta-communicatively), emblems mostly realize full speech-acts, for example the ‘okay gesture’ (Müller, 2010, 2014c). These gestural speech-acts can entail vocalizations or sometimes be paralleled by a verbal speech-act (sometimes this is the case with insults). Often they are used to replace a spoken language utterance. The three kinds of gestures operate as prototype categories, that is, they are not separated by sharp boundaries, their relations are dynamic. Throughout this paper the terms ‘singular gestures,’ ‘recurrent gestures,’ and ‘emblematic gestures’ are used as meta-terms to keep track of the different ways in which the term ‘gesture’ is used in the various frameworks discussed. Against this theoretical background, dynamic relations between gesture and sign are discussed. Such relations concern (a) historical change from gesture to sign, and (b) synchronic comparison of spoken and signed languages. The former includes lexicalization processes; the latter involves functional integration of gestures within a signed or spoken utterance (multimodal language use), and contact situations between spoken and signed languages (e.g., recurrent gestures used in Sign Language, or signing entering spoken languages).

The paper thus offers a framework against which the relations between gesture and sign can be systematically explored further by reconstructing the history of the discussion in the field of gesture studies and by formulating a position to illustrate how this can be achieved. The paper concludes by emphasizing the need for systematic cross-linguistic research on the multimodal use of language in its signed and spoken forms.

## Gesture-Sign Continua and Gesture as Utterance Visible Action: the Development of the Discussion in Gesture Studies

At least as far back as the Enlightenment we find reflections on the relation between gesture and sign (Copple, 2013; Kendon, 1975, 2002, 2004, chapter 3; Lane, 1979; Müller, 1998b, p. 51–53). One of the major reasons why this interest continues to motivate contemporary discussions is that the question of how gestures and signs relate to one another promises to provide insights into the nature and the origins of language itself (Kendon, 2002, 2008, 2014; McNeill, 2012, 2013; Wilcox, 2013).

A seminal moment in contemporary gesture studies was the publication of McNeill’s provocative paper “So you think gestures are non-verbal” (1985) with which he challenged the, at the time, dominant assumption that gestures were to be considered as *not* being related to language proper. Gestures were considered to be part of non-verbal communication, clearly and fundamentally different from language. Social psychologists Ekman and Friesen (1969) had presented a classification of non-verbal behavior, conceiving of hand gestures as illustrators to the stream of speech. Drawing on psycholinguistic evidence Feyereisen (1987),Butterworth and Hadar (1989) suggested a fundamental difference between gestures and speech (for an overview see Hadar, 2013). McNeill countered this position and engaged in a lively controversy with the then prevailing understanding of gesture as unrelated to language (McNeill, 1985, 1987, 1989). The importance of this discussion for gesture studies cannot be stressed enough. McNeill prepared the ground for a psychological and linguistic perspective on gesture, and showed that gesture is a highly valuable object of study for both psychologists and linguists. With the advent of Cognitive Science in the 1980s and 1990s, his model of gesture and speech as forming one integrated system opened the doors for linguists to study gesture. McNeill’s (1992) monograph *Hand and mind. What gestures reveal about thought* paved the way for gesture studies to emerge as a field. In McNeill’s psychological model, *gesture and speech are two sides of language*, each reflecting fundamentally different forms of thought (imagistic vs. propositional), but both indispensable because their categorical difference drives thinking as people are speaking. Kendon also adopted a critical stance toward the idea of gestures as forms of non-verbal communication. Already by Kendon (1972) had demonstrated the intimate link between gesture and speaking, and showed (in Kendon, 1980d) that gesture and speech are “two aspects of the process of utterance” (see also Kendon, 1983). Kendon’s work thus historically anticipated McNeill’s. This is reflected in the manifold references to Kendon’s work in McNeill’s early writings on gesture.

### Highlighting Commonalities: Gestures and Signs as Utterance Visible Actions (Kendon)

Kendon underlined the tight integration of gestures with speech in the process of utterance formation. In Kendon (1980c), he showed that gesticulation units are temporally aligned with ‘speech units’ and must be considered “an alternate manifestation of the process by which ‘ideas’ are encoded into patterns of behavior which can be apprehended by others as reportive of those ideas. It is as if the process of utterance has two channels of output into behavior: one by way of speech, the other by way of bodily movement.” (Kendon, 1980d, p. 218). In contrast to McNeill, however, Kendon’s interest in gesture early on included conventionalized gestures, so-called ‘emblems’ (Efron, 1941; Ekman and Friesen, 1969; Kendon, 1981, 1984), or ‘quotable gestures’ (Kendon, 1992) and, with his move to Australia, signed languages increasingly attracted his attention (Müller, 2007b). In the early 1980s, he published a series of papers on a kinesic system, a village sign language, employed by the Enga community in Papua New Guinea. Those papers offer a minute analysis of the formational properties, the semiotic functioning, and utterance construction of the Enga sign language (Kendon, 1980a,b,c). What began with an elaborate analysis of the primary sign language of the Enga in Papua New Guinea led to a broad study of alternate sign languages employed by Central Australian Aboriginal speech communities (Kendon, 1988b). In the same year as Kendon’s monumental work on Australian Aboriginal sign languages was published, a small book chapter appeared, which later inspired McNeill’s formulation of “Kendon’s continuum” (Kendon, 1988a, 2004, p. 104–106; McNeill, 1992, chapter 2). Kendon put forward arguments – historical, functional, and material (i.e., concerning the medium of expression) – in support of his view that “no sharp dividing line can be drawn between gesticulation that encodes meaning in a holistic fashion and gestures which, like so-called “emblems,” are not shaped on the spur of the moment but follow an established form within a communication community, or which like the signs in a sign language, can be shown to be structured systematically out of recombineable [*sic*] elements and which do indeed refer to meaning units of great generality, as do words.” (Kendon, 1988a, p. 134) Kendon considers both gesticulation and sign as *one gestural medium of expression* (Kendon, 2004, pp. 104, 307).

#### Historical Connections Between Gesticulation and Signs: Development as Lexicalization Process

By taking into account the full spectrum of gestures – including conventional and non-conventionalized kinesic forms – a historical process of sign formation from ad hoc created visual actions comes into view that can be described as a lexicalization process. In this process, gestures change over time, becoming increasingly stable, and may even develop into kinesic words, signs within a signed language. In his 1988 paper Kendon discusses different aspects of this process in a section entitled “Lexicalization of Gesture.” He begins by introducing emblems as “the functional equivalent of a complete speech-act,” a sbeing “standardized in form” and that “come to have meanings of much greater abstractness and generality” (Kendon, 1988a, p. 136). Concluding that “these forms are much more like words than anything we have heretofore considered,” he moves on to describe what happens when gestures become fully lexicalized:

“Gestures can become fully lexicalized when, for one reason or another, speech cannot be used for prolonged periods, but when nevertheless, all of the functions of spoken interchange are required. In these circumstances, where a spoken language is not available to create a context for gestural use, and where propositions must be exchanged as well as acts of interactional regulation, gestural units must be established that can serve, as words do, to refer to units of meaning that can be recombined to create complex signs with specific meanings.” (Kendon, 1988a, p. 136).

**FIGURE 1:**
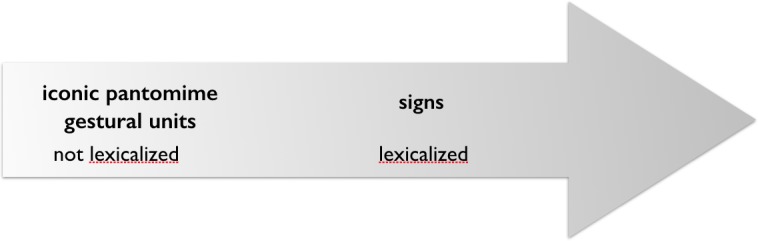
“Lexicalization of gesture”: historical development from gesture to sign.

This historical-developmental perspective on the gesture-sign relation is illustrated in **Figure 1**, representing the analysis of Kendon’s text by the author of this article.

At that point, ‘gesture’ for Kendon includes the entire range of kinesic forms and functions, from gesticulation as spontaneously created form “that encodes meaning in a holistic fashion”, to emblems and, notably, it *includes* signs. Emblems differ from gesticulation in that they have acquired a fixed-form meaning relation. Kendon describes them as “following an established form” and as such they are comparable to words. In linguistic terms, these gestures are lexicalized. Signs are described as being “structured systematically out of recombineable [*sic*] elements and which do indeed refer to meaning units of great generality, as do words.” (Kendon, 1988a, p. 134). Signs within signed languages may result from processes of lexicalization that start from the ad hoc creation of kinesic forms, ‘gesticulation’. Kendon’s analysis of how gestures may become like words thus includes the development of gestures into ‘kinesic words.’ In his 2004 book, such a historical-developmental perspective is discussed under the heading of “Iconicity, sign formation and the emergence of ‘phonology’.” Here an example from Scroggs (1981) is reported that describes spontaneous creations of gestures of a deaf boy (not trained in sign language) which started as iconic pantomime and became increasingly reduced in form as the boy was telling his story. Kendon describes the process as beginning with “an elaborate pantomime of mounting the cycle, starting it, revving it up, using hand motions to indicate the twisting of the throttle on the handlebar” (Kendon, 2004, p. 308). Over the course of the story the pantomime becomes reduced and abstracted to a hand motion. In other words, “the boy first created *representations* in gesture of the things he wished to refer to, and then he used *elements* from these representations as signs for these things.” (Kendon, 2004, p. 308; italics in the original). He then points out that it needs a speech-community for stable signs to develop and that the question of which elements are retained “in the transformation from elaborate depiction or enactment to a reduced sign-like gesture” depends upon their contrastiveness with “features of other gestures in the system” (Kendon, 2004, p. 308). Let me highlight two aspects of Kendon’s position as formulated here. First, Kendon speaks of ‘*sign-like gesture*,’ of ‘*gesture-signs*,’ and of ‘*other gestures in the system*’ using the term *‘gesture’* as a cover term for all kinesic forms of expression that are utterance dedicated visible actions used as utterances. Second, by describing the process of an emerging ‘gesture-sign’, he spells out a *historical continuity between spontaneously created, singular forms of gesture (or gesticulation or descriptive movement) and simplified, standardized, arbitrary forms (signs) that function as words in a kinesic system*. Note that arbitrariness is considered to be an outcome of a historical process of change.

**FIGURE 2:**
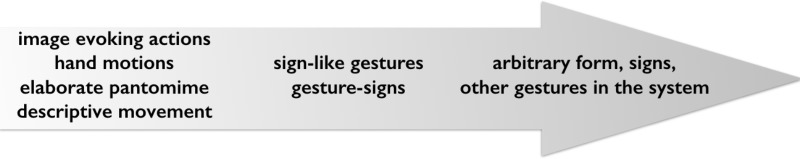
Visible actions as utterances: phases of historical development.

Kendon describes the phases of historical development as a path of transition that a spontaneously created gesture (gesticulation, singular gesture) may undergo on its way to an arbitrary sign (see **Figure 2**). He suggests that from “elaborate pantomime or descriptive movement sequence,” through simplification and “as a result of economy of action,” iconicity gets reduced (“is no longer apparent”) and “turns into an arbitrary form” under the ‘pressure’ to become a “distinctive form within a system of other forms” (Kendon, 2004, p. 308). Kendon not only points out that his view of the transitional process is grounded in his work on alternate and primary sign languages mentioned above, but also says, with reference to Klima and Bellugi (1979, chapters 1 and 3), Bellugi and Newkirk (1981), and Kyle and Woll (1985) that similar processes have been described in sign language studies *many times before*. In a nutshell, the argument Kendon unfolds is *an outline of the emergence of a kinesic language from spontaneous, singular forms of gestures, or from gesticulation*: “In this way, the visual representations and enactments for which the kinesic medium is so well adapted are transcended and a system of symbols that can operate in a quite abstract way is established.” (Kendon, 2004, p. 309).

Kendon’s ideas resonate with observations from (cognitive) linguistic research on historical changes of signs, here discussed under the label of *lexicalization and grammaticalization* processes. Wilcox (2005, 2007) describes routes from gestures to signed language with reference to American, Catalan, French, and Italian Sign Languages and with reference to historical documentations of gesture in the Mediterranean region. Several overviews of grammaticalization in sign languages have been offered (Wilcox et al., 2010; Pfau and Steinbach, 2011; Van Loon et al., 2014), and Janzen (2012) also discusses lexicalization. Kendon’s work can be considered an anticipation of this line of research and may also have been an incentive for it.

#### Functional Commonalities Between Gestures and Words in Spoken Languages

Already in that brief 1988a book chapter, Kendon brings in a second line of argumentation concerning the relation between gesture and sign: functional commonalities between gestures and words in spoken languages. From the point of view of communicative function, gestures can be used like words. This applies to all gestural forms, be they spontaneously created, holistic ones, or emblematic ones. Kendon already argues that gestures may be integrated in the vocal utterance, and then take over the function of a word. Many examples of semantic and pragmatic integration of a broad variety of hand-gestures in vocal utterances can be found throughout Kendon’s work. In the 2004 book, chapter 8 offers a series of ways in which gestures are deployed in the utterance; chapter 9 is devoted to “gesture and speech in semantic interaction”; chapter 10 shows how referential meaning of gestures is established and how this interacts with what is being said. Chapter 11 shows different forms of pointing gestures and how they work in conjunction with speech. Chapters 12 and 13 then discuss semiotic motivation and contexts of use of gestures with pragmatic functions and how they form gesture families. These chapters include accounts of gestures such as ‘precision grip’ gestures (otherwise known as the ring gesture) as well as open hand gestures and reconstructions of their functions: marking topic-comment or questions for the precision grip in combination with the open hand supine (Kendon, 2004, p. 262) are two examples. For all kinds of gestures, close analyses of their integration into the verbal utterance are given. One example used again in his 2014 paper is a speaker gesturally showing the size of cheese crates as he says “and they used to come in crates about as long as that” and outlining their shape while saying “and they were shaped like a threepenny bit at the ends” (Kendon, 2004, p. 166). Slama-Cazacu (1976) had already described this phenomenon as mixed syntax. More recently it has been described as simultaneous construction (Vermeerbergen and Demey, 2007), as multimodal grammatical integration (Fricke, 2013), as multimodal utterance (Ladewig, 2014a; Ladewig, in press), as composite signal (Clark, 1996; Engle, 1998); or as composite utterance (Enfield, 2009, 2013; Clark, 2016 for speakers; Janzen, 2017 for signers). This is how Kendon (2014) describes this kind of gesture-speech interaction: “In his words, thus, he talks about the length of the crates, and he describes the sort of shape they had, whereas his hand actions are now seen as showing the length and the shape. It is as if he is using his hands to draw sketches of the objects he is talking about and, by means of these sketches, he adds a kind of description, allowing, perhaps, the nature of the objects to be envisaged in a more precise way than the verbal description by itself might allow. The total meaning of what he is now saying is a product of an interaction between the meanings of his verbal phrases and the manually sketched illustrations that go with them. This is an example of what Enfield [34] has called a composite utterance.” (Kendon, 2014, p. 5) In short, gestures, understood as visible actions, can become functionally equivalents of spoken language ‘words,’ they can form composite utterances.

#### Commonalities of Kinesic Medium: Gesture and Sign Share the Medium of Expression

As a third commonality between gesture and sign, Kendon points out that both forms of expression are produced in the same kinesic medium: “Speakers’ uses of kinesic actions and signers’ uses of kinesic actions are cut from the same cloth” (Kendon, 2004, p. 324, chapter 15). Given Kendon’s intimate knowledge of primary and alternate sign languages and his work on conventionalized as well as non-conventionalized gestures, it is not surprising that material commonalities between gesture and sign come into view. In his 2004 book, an entire chapter is devoted to illustrating various ways in which ‘gesture’ and ‘sign’ can be understood as being ‘on common ground.’ Two issues are addressed: iconicity – involving sign formation and the emergence of kinesic phonology – and discourse construction. The discussion of iconicity and the emergence of kinesic phonology concerns the historical development of signs from spontaneously created gestures that we have dealt with above. Under the rubric of discourse construction, Kendon (2004, p. 310) discusses “features of the syntactic use of space and the use of ‘classifiers’ in sign language and describes examples of gesture use by speakers that seem very similar.” Regarding the use of space, he suggests that speakers employ space in much the same manner as signers do. One example he gives compares the spatial inflection of signs as described by Liddell (2003), where signers set up so-called surrogate spaces to which they then point to later on in their discourse. Kendon gives examples where a speaker does just the same thing, first when setting up a gesture scene and later on pointing to the location set up before gesturally. Concerning sign language classifiers he suggests that they have much in common with what has been described of techniques of representation in gesture studies: “In American Sign Language there is a high degree of consistency in how the various hand shapes for the different classifiers are used, and how the movement patterns are carried out when they are employed. However, this seems to be but a regularization of techniques that are widely used by speakers when using gesture for depictive purposes.” (Kendon, 2004, p. 318–319).

#### Utterance Visible Bodily Action: No Categorical Difference Between Gesture and Sign

Kendon offers three lines of argument in support of a view that sees no categorical difference between gesture and sign. He sees commonalities between gestures and signs with regard to historical, functional, and material aspects. In fact the commonalities between the two are considered so strong that he suggests giving up the term ‘gesture’ altogether and instead suggests replacing it by what he considers to be a more specific term: “utterance visible action” (Kendon, 2013, p. 7). He gives the following reasons for replacing the term gesture with “[…] utterance uses of visible bodily action”:

It is this that I shall call utterance visible action, and it corresponds to what is often referred to by the word “gesture.” However, because “gesture” is also sometimes used more widely to refer any kind of purposive action, for example the component actions of practical action sequences, or actions that may have symptomatic significance, such as self-touchings, patting the hair, fiddling with a wedding ring, rubbing the back of the head, and the like, because it is also used as a way of referring to the expressive significance of any sort of action (for example, saying that sending flowers to someone is a “gesture of affection”), and because, too, in some contexts the word “gesture” carries evaluative implications not always positive, it seems better to find a new and more specific term. (Kendon, 2013, p. 8).

In conclusion, Kendon’s position highlights c*ommonalities between different types of gestures and between gestures and signs*. In contrast to McNeill, he does not limit his account of the phenomenon to gesticulation (singular gestures), but includes conventionalized (recurrent) forms of co-speech gestures, emblematic gestures, as well as a thorough engagement with the analysis of sign languages.

Kendon (1988a) already suggested a bridge between gesture and sign against the backdrop of the historical development, functional and media specific commonalities:

I would like to suggest a different approach which, as I shall argue, can serve to link gesticulation with other kinds of gesturing, and which will also suggest that the gulf between presenting “content” in gesture and presenting it in “words” may not be as wide as it may now appear. At least I shall suggest a way in which a bridge may be built across that gulf. (Kendon, 1988a, p. 133).

The bridge Kendon offered a long time ago turned out to be not viable for McNeill and fellow psychologists, such as Singleton et al. (1995),Goldin-Meadow and Brentari (2017) or Emmorey (1999) (cf. also Kendon, 2000). Given their particular interest in gestures as windows onto thought, this is understandable. However, as we shall argue in the following section this comes at the cost of reducing the scope of gestural phenomena to those kinds of gestures that are spontaneously created, that are global-synthetic, holistic in the McNeillian sense, that are capable of revealing the ‘imagistic’ thoughts of speakers (McNeill, 1992), and that are able to “predict learning” (Goldin-Meadow and Brentari, 2017, p. 1). *In short, it limits the study of gesture to one type, namely to singular gestures.*

The gesture studies community received Kendon’s (1988a) reflections on the relation between gesture and sign in terms of a gesture-sign continuum through McNeill’s discussion of it and through his (1992) formulation of ‘Kendon’s continuum’ as an interpretation of the positions Kendon had formulated (1988a). Kendon, however, never liked the term and asked McNeill to not use it, which McNeill followed in his 2000 revision of the original continuum (see also Kendon’s discussion of it under the heading “Kendon’s continuum revisited” in Kendon, 2004, chapter 6, p. 104–106). Quite surprisingly, McNeill introduced the term ‘continuum,’ but then used it to highlight *discontinuities* between gesture and sign. While at first sight this contradiction might not seem obvious, it is what McNeill’s reflections on the different ‘gesture-sign continua’ come to conclude. In fact, based on the discussion of a potential continuum between gesture and sign, *McNeill diagnoses a categorical difference between the two, a difference termed ‘cataclysmic break’* in a co-authored paper by Singleton, Goldin-Meadow and McNeill in 1995.

### Highlighting Discontinuities: A Sharp Contrast Between Spontaneous Gestures and Socially Regulated Ones (McNeill)

It is puzzling. On the one hand, McNeill takes the radical counter position to psycholinguistic models on gestures by claiming that gestures are ‘verbal,’ meaning that they are an intrinsic part of language, rather than being *non*-verbal. On the other hand, he considers gestures as profoundly *different* from language. I propose that *this ‘difference’ is a consequence of a decision to restrict the concept of gesture to spontaneously used gestures*.

In McNeill’s work, the term ‘gesture’ refers only to singular gestures, gesticulation in Kendon’s terms. McNeill describes these gestural movements as being meaningful in a global-synthetic, holistic manner. McNeill (1992) clarifies that he uses the term “gesture” in this book specifically to refer to the leftmost, “gesticulation” end of the spectrum” (McNeill, 1992, p. 37). However, in ensuing discussions of the gesture-sign relation in the gesture studies community, the term ‘gesture’, originally referring to singular gestures, came to be used as a cover term, *pars pro toto*, to refer to gestures in general. This led to a tacit backgrounding of recurrent and emblematic gestures that are nevertheless very widely used along with speech (Müller, 2017). While the palm-up-open-hand (PUOH) gesture is conceived of as a singular gesture, metaphorically presenting the topic of discourse (McNeill, 1992, p. 14–15; see Cienki and Müller, 2014, for a critical discussion of metaphoric gestures; but also Parrill’s, 2008 critique of the conventional status of the PUOH-gesture), other recurrent gestures are not systematically discussed. For McNeill, conventional gestural forms (recurrent and emblematic) were not in his focus of interest since only spontaneously produced gestures (singular gestures) are psychologically interesting for him: they provide “an enriched view of the internal mental processes of speakers.” (McNeill, 1986, p. 108). They constitute a separate channel from speech and allow “a kind of triangulation onto the speaker’s mental representation” (McNeill, 1986, p. 108).

“A book about gestures and language.” This is how McNeill began his (1992) monograph. Crediting the discovery of the gesture-speech unity to Kendon’s observations on how gestures contribute to utterance construction, he had set out to develop a psychological theory of this relation. McNeill’s focus was always on singular gestures; as spontaneous creations of speakers they display individual ways of seeing the world. Singular gestures were viewed as images that are profoundly different from the conventional code of language, yet closely intertwined with speech:

The topic of this book was, specifically, gestures that exhibit images. With these kinds of gestures, people unwittingly display their inner thoughts and ways of understanding events in the world. These gestures are the person’s memories and thoughts rendered visible. Gestures are like thoughts themselves. They belong, not to the outside world, but to the inside one of memory, thought and mental images. Gesture images are complex, intricately interconnected, and not at all like photographs. Gestures open up a wholly new way of regarding thought processes, language, and the interactions of people.” (McNeill, 1992, p. 12).

It is important to go back to those very early formulations of McNeill’s theory of gesture and language, since they make it crystal-clear that he was interested in a specific kind of gestures, namely the individual, unique forms of gestures (i.e., the singular ones), because it is only these that allow insights into what he terms the imagistic side of language. This is the discovery he makes and he sets them apart from conventionalized gestures (recurrent and emblematic) that scholars from Antiquity to present times have dealt with: “None of these early investigators, however, considered the spontaneous gestures accompanying speech that are the chief focus of this book” (McNeill, 1992, p. 3). It is in the dialectic of singular gestures as ‘images’ and speech as a system of codified forms that McNeill sees two different forms of thought:

They [singular gestures] are closely linked to speech, yet present meaning in a form fundamentally different from that of speech. My own hypothesis is that speech and gesture [singular gestures] are elements of a single integrated process of utterance formation in which there is a synthesis of opposites modes of thought–global-synthetic and instantaneous imagery with linear-segmented temporally extended verbalization. Utterance and thought realized in them are both imagery and language (McNeill, 1992, p. 35).

This means, when formulating his hypothesis concerning speech and gesture as “elements of a single integrated process of utterance formation” and characterizing this process as a “synthesis of opposites modes of thought–global-synthetic and instantaneous imagery with linear-segmented temporally extended verbalization,” singular gestures are being described as revealing the imagistic side of thought while speech reveals the linear-segmented form of thought. Put differently, what McNeill is interested in are the insights into ‘imagistic’ forms of thought that only the individual, spontaneously created gestures can offer.

This explains why *conventional (recurrent and emblematic) gestures are not in the scope of McNeill’s interest*. In his approach to gesture, conventional gestures switch sides, *they become like language and thus lose the unique capacity of opening up a window onto a speaker’s mind*. Conventional gestures are thus *qua definition* excluded from McNeill’s use of the term gesture. A continuum between the two thus cannot come into view, because these forms are excluded pre-hoc (as with emblems), or are not considered as being conventional (see above), which at least for the ‘ring gesture’ is undebatable even when used as a pragmatic co-speech gesture (Neumann, 2004; Müller, 2014c). The importance of the distinction between singular gestures and conventional recurrent and emblematic ones for McNeill is immense. He devotes the second chapter of his book to a substantiation of the fundamental difference between spontaneous gestures and codified signs:

The focus of this book is on spontaneous and idiosyncratic gestures (…) but it is useful to begin (…) with the more language-like gestures that constitute sign-languages. These are signs organized into true linguistic codes. *We benefit in this way from the sharp contrast that we can draw between the spontaneous and the socially regulated kinds of gesture*. (McNeill, 1992, p. 36; emphasis added).

**FIGURE 3:**
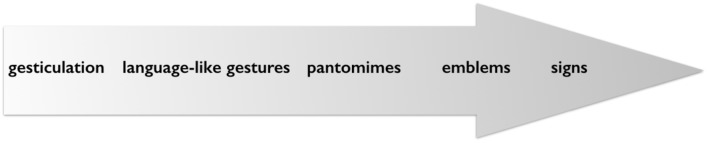
McNeill’s initial formulation of ‘Kendon’s continuum (adapted from McNeill, 1992, p. 37).

The sharp contrast drawn by McNeill concerns singular gestures on the one hand, and recurrent and emblematic gestures on the other. In the formulation of this contrast, historical development and functional aspects are collapsed and put along one continuum, discussed broadly as gesture’s relation with speech (**Figure 3** adapted from McNeill, 1992, p. 3): “As we move from left to right: (1) the obligatory presence of speech declines, (2) the presence of language properties increases, and (3) idiosyncratic gestures are replaced by socially regulated signs.” (McNeill, 1992, p. 37) Note that here the term ‘gesture’ is used as a cover term to include spontaneous and conventional forms: gesticulation, language-like gestures, pantomimes and emblems.

**FIGURE 4:**
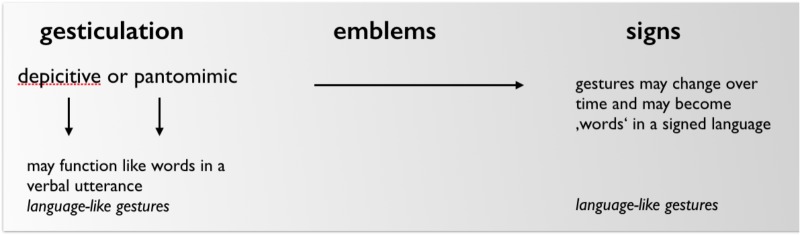
Functional and historical aspects included in Kendon (1988a).

**FIGURE 5:**
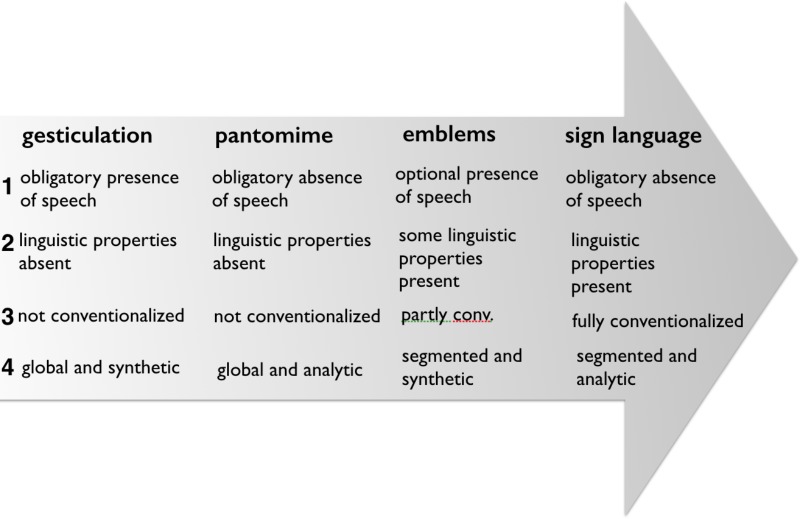
McNeill’s (2000) expanded version of the gesture-sign continuum (adapted from McNeill, 2000, p. 1–10).

What McNeill does is to put the functional integration of singular gestures into a verbal utterance (e.g., mixed syntax, or linear integration of ‘language-like’ gestures) on the same level as the historical development from gestures, to emblems, to signs. He thus blends the functional argument with the historical one. Moreover, in Kendon (1988a) gesticulation, language-like gestures and pantomimes are not described as alternatives. For Kendon gesticulation *includes* depictive as well as pantomimic gestures, and both can be used in a language-like function (**Figure 4**). But commonalities regarding gesture and sign as expressive medium are excluded from the continuum in McNeill. Although McNeill later published a revised and expanded version of the continuum (McNeill, 2000), this blurring of historical and functional perspectives and the exclusion of commonalities concerning the kinesic medium of expression is maintained. Four aspects of the gesture-sign continuum are discussed separately: (1) the relationship to speech, (2) the relationship to linguistic properties, (3) the relationship to conventions, and (4) the character of semiosis (McNeill, 2000, p. 1–7). **Figure 5** (adapted from McNeill, 2000) gives an overview of the changes along the continuum. Here again the term gesture is used in a broad sense to include non-conventional as well as conventional gestures (gesticulation, pantomime, emblems). **Figure 5** shows an overview of the four sub-continua.

McNeill suggests that, as one moves from gesticulation to sign, the obligatory presence of speech decreases (emblems and pantomime switch places here), linguistic properties (in terms of segmentation) increase, the character of semiosis changes from global-synthetic to segmented-analytic, and with conventionalization come emblems and signs. This description actually could be read as describing the historical processes of gesture change that both Kendon (1988a) and sign language studies describe as lexicalization (Janzen, 2012, see above and below). McNeill, however, establishes a clear-cut dividing line between gesture and sign as if processes of increasing conventionalization were impossible. Yet this is precisely what Kendon keeps pointing out. McNeill’s continuum thus establishes a sharp dividing line between non-conventional and conventional forms *qua* an implied definition. *Instead of a gesture-sign continuum a categorical distinction between gesture and sign is established.*

But why are the continua so important for McNeill that he reconsiders them and even expands his exposition? The answer is that they are vital in defining the scope of phenomena covered by his psychological Growth-Point model. Only those forms of gesture that show an obligatory presence of speech, that have no linguistic properties, that are not conventionalized and whose meaning is constituted in global and synthetic manner (e.g., singular gestures) are able to reveal the imagistic side of thought. It is important to bear in mind that the concept of gestures as images is a rather idiosyncratic position of McNeill. Not only does it employ the term ‘image’ in a rather unelaborated manner, but it also backgrounds the fact that gestures are first and foremost *movements* of the hands often engaged in as-if actions and not images (Kappelhoff and Müller, 2011; Müller, 2014b, 2016, 2018). A concept of gesture as image disregards the practical engagements of the hands in mundane practices (cf., Streeck, 2009, 2013, 2017) and the way manual actions ground meaning of gestures (Müller, 1998a,b, 2004, 2010, 2014b, 2016, 2017; Kendon, 2004). For McNeill’s model of thought processes, the *difference* between imagistic and propositional thought remains as fundamental as does the *difference* between spontaneous and conventional gestures, e.g., between singular and recurrent or emblematic gestures. It is the dialectic between imagistic and propositional forms of thought in the mental Growth-Point that, following McNeill, are said to drive thinking processes forward. When singular gestures become language-like, they change sides and also imprint thought with propositional structures which are characteristic of a conventionalized system of codified signs. That is, conventionalized gestures (recurrent and emblematic) are not in the scope of interest in McNeill’s concept of gesture because only the individual spontaneous (singular) gestures of speakers reveal the hidden imagery of thought.

Such a limitation of the scope of phenomena under scrutiny is absolutely legitimate as long as it is dealt with explicitly, which McNeill very clearly does. It is very productive and even necessary for experimental studies. It is not helpful, however, in elucidating historical perspectives of gesture change, commonalities between gestures and signs that concern their shared kinesic medium of expression, or the roles different types of gestures, including conventional gestures recurrently co-occurring with speech, play in the construction of multimodal utterances (Ladewig, 2014a,b,c; Müller, 2017; Ladewig, in press). Against this background, the postulation of a categorical divide or a ‘cataclysmic break’ between gesture and sign appears as deliberate exclusion of phenomena. This is perfectly legitimate to underline a specific aspect of gesture (as revealing spontaneous gestural forms of conceptualization, for example), or in an experimental design. It can, however, *not* be considered a response to the question of the gesture-sign relation in general, since it excludes recurrent and emblematic gestures, which are, nevertheless, extremely widespread aspects of multimodal utterance construction (Bressem and Müller, 2014a,b, 2017). The exclusion blurs potential ‘continuities’ that cannot come into view, since they fall outside the scope of the phenomena investigated. It also excludes reflections concerning the material commonalities between gesture and sign, relating to the medium of expression, both historically and when gestures are used by signers. Against this backdrop, the cataclysmic break recently restated by Goldin-Meadow and Brentari (2017) must be considered *an ‘artifact’ of definitions*.

### A ‘Cataclysmic Break’: ‘Imagistic’ Gesture and Categorical Sign (Goldin-Meadow and Brentari)

McNeill’s position was formulated in the early nineties. It still informs the discussion on the relation between gesture and sign. Current discussions continue the above blurring of aspects of the gesture-sign relation that was an understandable consequence of McNeill’s theory of gesture and language. In a recent paper, Goldin-Meadow and Brentari (2017) present a detailed overview of the state of the art concerning the relation between gesture, sign and language. It is a strengthening of the McNeillian position against a Kendonian view of that relationship. Goldin Meadow and Brentari’s paper addresses the question: “How does sign language compare to gesture, on the one hand, and to spoken language on the other?” It tackles these questions strictly from a McNeillian point of view and “conclude that signers gesture just as speakers do. Both produce imagistic [singular] gestures along with more categorical signs or words.” (Goldin-Meadow and Brentari, 2017, p. 1). The authors compare gesture-speech and gesture-sign systems as temporally co-existent systems, and apply the McNeillian concept of gesture as spontaneously created and ‘encoding’ meaning in an idiosyncratic, global-synthetic, holistic manner. In other words, they focus on singular gestures. Goldin-Meadow offers a psychological motivation for using the term gesture only for ‘imagistic,’ spontaneous forms of gesture: “we argue that making a distinction between sign (or speech) and gesture is essential to predict certain types of learning” (Goldin-Meadow and Brentari, 2017, p. 1). The authors conclude that “a full treatment of language needs to include both the more categorical (sign or speech) and the more imagistic (gestural) components regardless of modality and that, in order to make predictions about learning, we need to recognize (and figure out how to make) a critical divide between the two” (Goldin-Meadow and Brentari, 2017, p. 2). It is crucial that Goldin-Meadow and Brentari make their definition of gesture explicit. What they do not make explicit, however, is that this excludes conventional co-speech gesturing once again, as in McNeill, *qua an implied definition*. As a consequence, what cannot come into view is a dynamic process of gesture change in which spontaneously created gestural forms may increasingly stabilize, and in which hybrid forms may emerge, such as is the case in recurrent gestures regularly employed by speakers across different discourse types (Müller, 2017, see also Kendon, 2004, p. 104). *The “critical divide” thus is a result, as in McNeill’s work, of the definitional limiting of gesture to singular gestures and the resulting disregard of conventional forms of co-speech gesture.* With regard to defining the scope of behaviors relevant for their work, Goldin-Meadow and Brentari apply the McNeillian framework and thus their position differs fundamentally from Kendon’s.

The different definitions or concepts of ‘gesture’ have important implications for the different concept of language the authors favor. While for Goldin-Meadow and Brentari, the simultaneity of spontaneous gesture with vocal and with signed languages shows the critical divide between what is ‘gesture’ and what is ‘language,’ for Kendon, the simultaneity of the full spectrum of ‘visible bodily actions’ with spoken and with signed languages indicates that the traditional concept of language is too narrow and should include the full range of visible bodily action as a close interrelation of different ‘semiotic systems.’ Kendon’s alternative to the concept of a sharp boundary between gesture and sign is the broadening of the concept of language to include different modalities and a flexible interrelation of different semiotic systems (Kendon, 2014, p. 3).

Goldin-Meadow and Brentari elaborate on the McNeillian position and highlight the endpoints of McNeill’s continuum to illustrate a discontinuity between gesture and sign. As a consequence, differences are maximized and the relation between singular gestures and signs is constructed as categorically distinct, as separated by a ‘cataclysmic break.’ This is how Singleton et al. (1995) formulated it in the title of a chapter: “The cataclysmic break between gesticulation and sign: Evidence against a unified continuum of gestural communication.” Here experimental evidence is offered to reject the idea of a continuity along the gesture-sign continuum that McNeill (1992) had attributed to Kendon’s (1988a) analysis. In a psychological experiment, speakers were placed in one of two conditions: describing previously seen events with and without speech. In the suppressed speech condition, the appearance of the gestures changed; they became more elaborate, more discrete. In the authors’ view, they became more language-like, more segmented, forming ordered strings. Goldin-Meadow and Brentari (2017, p. 9) summarize the results in the following way: “The gestures without speech immediately took on sign-like properties—they were discrete in form, with gestures forming segmented word-like units that were concatenated into strings characterized by consistent (non-English) order.” Notably, the authors attribute the change uniquely to the fact that spoken language was suppressed, and gestures had to carry the full communicative burden. The basic argument was to show that once an individual had to communicate only manually, without making use of spoken language, the appearance of gestures changed *instantaneously*, and from one moment to another an individual speaker ‘invented’ signs. This might be why Goldin-Meadow (2015) characterized these gestures as ‘silent gestures’ and more recently as ‘spontaneous signs’ (Goldin-Meadow and Brentari, 2017). The implications drawn from this experiment are far-reaching and re-state a categorical divide between gesture and sign:

(1) There is a qualitative difference between hand movements when they are produced along with speech (i.e., when they are gestures) and when they are required to carry the full burden of communication without speech (when they begin to take on linguistic properties and thus resemble signs); and (2) this change can take place instantly in a hearing individual. Taken together, the findings provide support for a categorical divide between these two forms of manual communication (i.e., between gesture and sign), and suggest that when gesture is silent, it crosses the divide (see also Kendon, 1988a). In this sense, silent gesture might be more appropriately called “spontaneous sign” (Goldin-Meadow and Brentari, 2017, p. 9).

If, however, the term gesture is reduced to singular gestures, then once again, the divide is caused by the definition. By deliberately excluding conventionalized co-speech gestures and by restricting the focus of analysis to a very specific experimental setting, gradual processes of change between spontaneous and conventional forms as they may happen in ordinary language use (Müller, 2017; see also Ladewig, 2010, 2011, 2014c) cannot come into view because of (a) *the definition of the term gesture*, and (b) *the restrictions of the experimental setting*. Consequently, conclusions drawn from this specific experimental condition are not viable for making claims beyond this specific experimental condition. Gesture change as an historical process can thus not come into view. This also holds for the various forms of functional integration of gestures within utterances as observed under naturalistic circumstances of language use. They are excluded, because they are not considered an object of inquiry.

Moreover, the interpretation of this experimental condition suggests that all it needs for language-like gestures to emerge is to suppress vocal language. However, no individual can produce a language. *What is needed for a language to appear is understanding, the reflexivity and intersubjectivity of meaning shared within a moment of discourse or across a community of speakers/signers*. Observing strings of ‘silent gestures’ under experimental conditions does not tell us whether they are understood by a conversational partner, or whether they function within a speech community. As a consequence, it does not tell us whether they *functionally* replace speech as a socially shared communicative system.

Goldin-Meadow and Brentari’s claim that all it needs for gesture to cross the ‘divide to language’ is to suppress spoken or signed language does not hold in light of observations concerning schematizations and generalizations of gestures and emergent sign described above. Kendon’s descriptions of processes of form reduction and generalization of meaning happen very quickly in emergent signing and are extremely frequent in naturalistic contexts of multimodal language use (Kendon, 2013; see also Müller, 2017). Those processes obviously only can come into view under the condition (a) that the concept of gesture is not restricted to singular gestures, but includes singular, recurrent, as well as emblematic gestures, and (b) that gestures are studied across a broad range of different naturalistic discursive contexts.

Having introduced the idea of ‘silent gestures’ as indicators of a so-called cataclysmic break in the gesture-sign continuum, Goldin-Meadow and Brentari expand their view from the experimental setting to culturally shared repertoires of codified gestures arguing that those are the same kind of ‘silent gestures’ as the ones observed under the experimental condition described above. The common ground for those two very different forms of gesture *usage* is that they are said to be employed in the absence of speech. Put differently, the authors move directly from spontaneous co-speech gestures as produced under experimental conditions to codified sign systems. As a consequence, processes of gradual change cannot be uncovered, because precisely those kinds of gestures and those gestural usage contexts that could show such a gradual change are excluded.

However, the famous saw-mill gestures, monastic sign languages, or Aboriginal sign languages are all historical products of a communication community, they have evolved over time and have developed conventionalized repertoires of fixed form-meaning pairings, and a word-order (Kendon, 2004, chapters 14, 15; Kendon, 2013, p. 18). Kendon (2013) discusses these processes under the heading of “When utterance visible action is the main utterance vehicle.” He points out that historical processes of sign formation have been widely discussed in sign language research that involve a historical and gradual transition from more complex kinesic enactments to more schematized ones, and this transition presupposes the social sharing of kinesic forms. Under naturalistic conditions of language use, it is through the back and forth between co-participants that schematization of forms and generalization of meaning happens (see also McNeill and Sowa, 2011):

To represent a meaning for someone else (and also, I think, to represent it for oneself), one resorts to a sort of re-creation. As if, by showing the other the thing that is meant, the other will come to grasp it in a way that overlaps with the way it is grasped by oneself. As these representations become socially shared, they rapidly undergo various processes of schematization. In consequence they are no longer understood only because they are depictions of something but also because they are forms which contrast with other forms in the system, acquiring the status of lexical items in a system. (Kendon, 2013, p. 18).

Rather than appearing instantaneously within one individual, codified kinesic languages are thus products of a historical process of language formation that critically depends on a community of users, be they engaged in a dyadic encounter or as members of larger communicative ensembles.

Although such a historical perspective on the gesture-sign relation clearly *contradicts* the discontinuity assumption of a ‘cataclysmic break,’ Goldin-Meadow and Brentari do mention processes of historical change: “Although the gesture forms initially are transparent depictions of their referents, over time they become less motivated, and as a result, less conventionalized, just as signs do in sign languages evolving in deaf communities (Frishberg, 1975; Burling, 1999)” (Goldin-Meadow and Brentari, 2017, p. 9). It is a logical consequence of their definition of gesture that, after conceding this historical process, the authors nevertheless come to the conclusion that “in all of these situations, the manual systems that develop look more like silent gestures than like the gestures that co-occur with speech.” If the term gesture refers to singular gestures only (idiosyncratic gestures in McNeill’s terminology and understanding) produced under experimental conditions, then (a) spontaneous processes of schematization and abstraction of singular gestures cannot come into view, because naturalistic conditions of use are not considered in which they happen very frequently, and (b) hybrid gesture forms that involve stabilized and non-stabilized formational aspects cannot come into view because recurrent and emblematic gestures are excluded qua definition (Müller, 2017).

Once again, the claim of a critical divide between gesture and sign is the result of a deliberate decision of (a) excluding conventional (co-speech and co-sign) gestures, and (b) experimental settings (which implies the exclusion of linguistic analysis of gesture-speech integration in its ordinary forms and contexts). Moreover, it implies a static and monadic concept of language as being either present or not, and as something that can appear ‘instantaneously’ within one individual.

Goldin-Meadow and Brentari also point out that ‘silent gestures’ in contrast to alternate sign languages, do not follow English word order. An explanation to this might be the fact that silent gestures are in fact, not like language at all. Because they lack the social sharing across a community of speakers and across the variable contexts of everyday life. The forms and repertoires of so-called ‘silent gestures’ never actually leave the experimental context, they are not taken up, changed, altered, adapted to other contexts of use, and they are never employed for complex communicative purposes. Thus, silent gestures do not have a chance to develop, simply because they are not used recurrently by a community of speakers under ordinary conditions of everyday life. Only if this happens, can we really see if English word order would be instantiated in gestures or not. For Goldin-Meadow and Brentari, these are the grounds on which they “argue that there are strong empirical reasons to distinguish between linguistic forms (both signed and spoken) and gestural forms,” and “that doing so allows to us make predictions about learning that we would not otherwise be able to make.” (Goldin-Meadow and Brentari, 2017, p. 2) Against our critical reading of the arguments, the empirical grounds presented by Goldin-Meadow and Brentari appear in fact rather weak. They rest upon (a) a restricted concept of gesture, (b) a highly specific experimental condition, and (c) a static and narrow concept of language. In fact, the narrow focus of their claims is asserted by the authors themselves, namely, by linking it to the possibility of making predictions about learning from singular gestures.

Clearly, adopting a narrow focus is legitimate for psychological research, and this is what they state in the above quotation, but three problems remain: (1) it does not tell us anything about how speakers and signers use recurrent and emblematic gestures; (2) it is not suited for proving a historical divide between singular, recurrent, and emblematic gestures and signs; (3) it does not tell us anything about functional commonalities between gestures and spoken or signed words.

Summing up, Goldin-Meadow and Brentari’s position comes with a strong reduction of the scope of relevant behaviors included under the rubric of gesture, which clearly is crucial for psychological reasoning. For communicative, linguistic, anthropological, semiotic, and functional analyses of gestures this appears as a deliberate and artificial boundary which excludes qua definition hand movements in their full scope of phenomenological appearance in naturalistic settings. The validity of these findings for understanding relations between gestures and signs with respect to their communicative and linguistic functions must, therefore, be considered rather weak.

If the full spectrum of co-speech gesture is not considered, that is, *conventionalized* co-speech gestures are excluded, then gradual processes of change in the gestural medium of expression cannot come into view. What may happen if they are considered is the subject of the second section of this paper.

## Beyond the Cataclysmic Break: Dynamic Relations Between Gesture and Sign

In this section, a plea is made for conceiving of relations between gesture and sign as dynamic. This shift involves a broad definition of the term gesture, and a consideration of gesture-sign relations from two different perspectives: the historical dynamics of gesture change, and a comparative view of two ‘multimodal’ languages in contact (for example, Deutsche Gebärdensprache, DGS, German Sign Language, and spoken German). The comparative perspective includes dynamic relations between gestures and signs within and across languages. It is informed by Kendon’s multiple observations on the relation, as presented above, and it considers a discussion of gesture-sign continua as initiated by McNeill as vital for the discussion. The position sketched out here is thus informed by both lines of research in gesture studies. It does, however, not follow the assumption of a critical divide or a cataclysmic break between gesture and speech. Instead the relation between the two expressive modalities is considered as a dynamic one with regard to three different aspects: (a) historical development, (b) within, and (c) across spoken and signed languages. This position starts from a concept of language as inherently multimodal (Müller, 2007a, 2008). It is in line with Janzen (2017) who considers multimodality “a general characteristic of language, with composite utterances as instantiations of multimodality” (Janzen, 2017, p. 519) Furthermore, it is based on a linguistic perspective of multimodal language use (Müller, 2007a; Müller et al., 2013a; see also Ladewig, 2014a; Bressem and Müller, 2017; Ladewig, in press). It takes the analysis of multimodal language as it is used across contexts as a basis for exploring manifold possible relations between gesture and sign (Müller, 2009; Bressem et al., 2018). This includes the analysis of gestures and signs across different naturalistic but also experimental contexts. It presupposes a close semiotic, interactional, and linguistic analysis of all the gestural forms we observe ‘in the wild’ (Müller, 2010, 2016, 2017; Bressem et al., 2013, see also Mittelberg, 2013, 2014; Mittelberg and Evola, 2014; Mittelberg and Waugh, 2014) and the multitude of ways in which they are integrated with speech or sign creating simultaneous structures (Vermeerbergen and Demey, 2007), composite utterances (Enfield, 2009, 2013; Janzen, 2017), gesture-speech ensembles (Kendon, 2004), or multimodal utterances (Ladewig, 2014a; Ladewig, in press). It also starts from a broader notion of the term gesture than the one suggested by McNeill and Goldin-Meadow.

### Spelling Out the Concept of Gesture

Spelling out one’s concept of gesture, even if an absolutely watertight definition remains unattainable, is crucial since it determines the scope of relevant behaviors that become relevant to empirical investigation and theoretical reflection. Moreover, it also explicates the theoretical framework within which a given assumption, research, proposal, and claims concerning gesture are formulated. As a consequence, the spectrum of phenomena covered by the claims is made explicit.

Although I agree with Goldin-Meadow and Brentari “that a full treatment of language needs to include both the more categorical (sign or speech) and the more imagistic (gestural) components regardless of modality (see also Kendon, 2014)” (Goldin-Meadow and Brentari, 2017, p. 2), I do not, agree with the assumption that gestural equals imagistic, nor that there is a clear-cut boundary between categorical and gestural.

From a usage-based and interactional point of view, gestures are meaningful body movements whose meaning is grounded in embodied experiences that are dynamic and intersubjective, and not at all like images (Müller, 2017; Müller and Kappelhoff, 2018). Put differently, I advocate an understanding of gestures as deliberate expressive movements (Kappelhoff and Müller, 2011; Müller, 2014a; see also Kendon, 2004, chapter 2). Semiotically, gestures are motivated by as-if actions, enactments of movement, or object representations (Müller, 2014b, 2016, 2017, 2018; see also Mittelberg, 2013, 2014). Gestures show degrees of conventionalization, understood as sedimentation of experiential frames (Müller, 2017). Degrees of conventionalization may range from none to partially to fully conventionalized. These different degrees are reflected in the terms “singular, recurrent, and emblematic gestures.” Although the terms suggest categorical differences, these are not implied. Rather, we find different forms of hybridity between them (Müller, 2017).

An explication of the term ‘gesture’ helps to improve clarity in the discussion concerning the relation between gesture and sign. I favor using the term ‘gesture’ over the replacement ‘utterance visible action’ suggested by Kendon because, although this phrase was introduced to broaden the scope of behaviors under consideration, I suggest that, in fact, it narrows it down. Moreover, it implicitly establishes a specific theoretical focus. If ‘utterance visible action’ is applied *semiotically*, that is, if it refers to the motivation of gestures, then this implies that gestures are *only* grounded in actions of the body. This excludes gestures that are enactments of movement and it excludes hybrid gestures, where some facets of a gestural movement, may be used to express aspects of meaning that are independent from the type of gesture. An example would be the deictic orientation of a horizontal ring gesture toward an addressee in contexts of expressing agreement and preciseness of an argument made by an interlocutor (a gestural expression of ratification and precision) (Müller, 2017). In that case the ring shape would be motivated by an as-if action of grasping while the movement toward the interlocutor is a deictic movement. Another case is the possibility to express aspectuality, understood as temporal contour of events, with a bounded (perfective) or an unbounded (imperfective) movement quality of a gesture (Müller, 2000, 2018). In a cross-linguistic study on aspectuality in Russian, French, and German significant correlations between perfective and imperfective past tense and bounded and unbounded gestural movement qualities were found for French speakers (Cienki and Iriskhanova, 2018). This perspective on the verbo-gestural expression of aspect goes along with a proposition made by linguists from various traditions (Behaghel, 1924; Holt, 1943; Croft, 2012), who proposed that verbs in the perfect(ive) tense characterize events as bounded in some way, as opposed to those in the imperfect(ive). Kinesically, boundedness was determined as pulse of effort, and unboundedness as more controlled movement, without a clear pulse of effort (Müller, 1998b, 2000, 2018; Boutet, 2010). We found that French speakers used significantly more “bounded” gestures, when they used the perfective tense (Passé compose). With the imperfective tense (Imparfait) the pattern was reversed. Speakers used more unbounded gestures (Cienki and Iriskhanova, 2018).

Furthermore, if ‘utterance visible action’ is understood as semiotic motivation in bodily actions only, then the concept would exclude gestures that are semiotically re-presentations of objects, when the hand becomes a body sculpture of a picture or a window or a piece of paper (Müller, 1998a,b, 2014b, 2016).

If, on the other hand, ‘utterance visible action’ refers to ‘action’ as a *theoretical concept*, then this implies a praxeological theory of communication (e.g., Streeck, 2013), which is an extremely important move in gesture theory interesting with far-reaching theoretical implication for gesture and speech as multimodal interaction, but it also implies a narrowing down of the theoretical focus more than the term gesture as currently employed in gesture studies. It is one of the strengths of the field of gesture studies that the term gesture allows for different theoretical frameworks to be applied and accordingly for different definitions and foci on gesture. As long as the respective concepts of gesture are spelled out explicitly, misunderstandings can be avoided and a critical discussion between the different positions fostered. To accurately gauge claims about gesture in Goldin-Meadow’s and McNeill’s work, it is important to know that the term gesture in their studies refers *only* to singular gestures (idiosyncratic gestures in the McNeillian sense). Conversely, to assess claims about gesture in Kendon, Streeck, or Müller’s work, it is equally important to know that here the term gesture involves a broader spectrum of bodily behaviors, including singular, recurrent and emblematic gestures. Kendon’s recent plea for the notion of ‘utterance visible action’ obviously includes all of those and even “actions performed in the course of creating utterances in sign language” (Kendon, 2013, p. 8).

The following sections will illustrate how such a broad concept of gesture reveals dynamic relations between gesture and sign that a narrow one excludes qua definition.^1^ The discussion and the claims made concerning the relation between gesture and sign are structured around two perspectives: a historical and a comparative one (within and across spoken and signed languages). Adopting such a broad concept reveals dynamic relations to be a fundamental characteristic of gesture.

### Gesture Change: Historical Dynamics From Gesture to Sign

**FIGURE 6:**
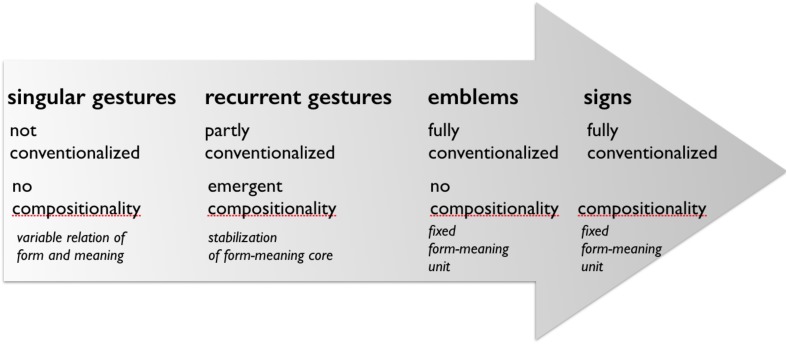
Gesture change: historical development from gesture to sign in terms of degree of conventionalization and compositionality as an emergent hybridization.

When taking into account the full spectrum of gestural expression, it becomes clear that non-stabilized, somewhat stabilized, and fully conventional gestural forms may be employed by language users. These forms are not sharply separated from one another as discrete categories. Rather, they can be thought of as arranged on a continuum from individually improvised forms to forms that are fully conventionalized. This is in line with Kendon’s (2014, p. 6) position: “These (and other) representational practices […] are widely shared and are subject to varying degrees of social conventionalization. Some forelimb utterance actions may become so standardized that they acquire meanings that may be glossed with stable verbal expressions (often known as ‘emblems’ [39]), and, as such, are sometimes used as substitutes for spoken words in some contexts. In this case, we have something comparable to a lexical sign in a sign language” (Kendon, 2014, p. 6). However, in addition to Kendon’s sketch, we include recurrent gestures as an intermediate and hybrid form of gesture that is placed between singular and emblematic gestures with regard to conventionalization (Ladewig, 2010, 2011, 2014b,c; Müller, 2010, 2017). This developmental position between gesture and sign critically rests on their material commonality as a medium of expression. **Figure 6** systematizes a potential historical dynamics based on the degree of conventionalization and compositionality as an emergent feature. Note that historical development from gesture to sign may start with any of those three types of gestures.

**Figure 6** is inspired by McNeill’s continuum (3) (McNeill, 2000, p. 4) and reflects an understanding of conventionalization as a successive, dynamic process of constant change (see also Gullberg’s, 1998 discussion of the continuum) and agrees with Gullberg’s refined systematics of the continuum’s left side, where she points out that the spontaneous forms of gesture (gesticulation) in fact entail a range of different varieties and includes, for instance, depictive as well as pantomimic forms (Gullberg, 1998, chapter 3). Singular gestures are considered to be gestures that are not conventionalized, that show a variable relation of form and meaning, and that are not compositional.

**Figure 6** thus illustrates conventionalization as a gradual process (McNeill describes emblems as partly conventionalized, see also Gullberg, 1998, chapter 3), and introduces recurrent gestures: “By merging conventional with idiosyncratic or other conventional elements, recurrent gestures occupy a place between spontaneously created (singular) gestures and emblems as fully conventionalized gestural expressions on a continuum of increasing conventionalization” (Müller, 2017, p. 278). Examples of recurrent gestures are gestures that build families in the Kendonian sense, and that come with a stable form-meaning pairing (Bressem and Müller, 2014a,b, 2017; Ladewig, 2014b,c). A consequence of such conventionalization processes is that they affect gestural forms and functions gradually, and involve *hybridization* of spontaneous and more stabilized gestural forms and functions (Müller, 2017). From such a perspective, compositionality is a consequence of a process of decomposing holistic form-meaning units into stabilized formational cores with a shared semantic theme (to employ Kendon’s terms here, see also Kendon, 2004, p. 104). Formational features that are not involved can be used to express local meanings spontaneously (position in gesture space is often used in this way), or they can include other stabilized formational features. Recurrent gestures thus show emergent forms of compositionality. In emblematic gestures all formational features tend to be stabilized, and in that sense they are not compositional. Signs, however, are conventionalized and compositional, as in the case of spatial verbs described above. The compositionality of signs might be a consequence of accommodation and assimilation within a linguistic system, but this issue needs further exploration, at least as far as comparative gesture-sign language studies point of view is concerned. Recurrent gestures differ from emblems not only regarding their hybridity, but also regarding their functions (Müller, 2010, 2017; Ladewig, 2014b). Recurrent gestures function meta-communicatively and are thus inextricably connected with speech, emblematic gestures are fully conventionalized and typically function as complete speech-acts; although they often include vocal elements, they are more independent from the co-presence of speech than recurrent gestures (Teßendorf, 2013).

When considering the full range of gestural phenomena, which, as we have seen, was Kendon’s position early on when he argued that gestures may lexicalize, it is possible to see that gestures are affected by processes of conventionalization, which – as in spoken language – are gradient and not at all sudden. Those processes go along with tacit agreements of a community of language users and the changes involved concern gestural forms *and* functions that emerge *from*, and change *with*, language use. In sign language research, such processes of change have been described in terms of lexicalization and grammaticalization, that is, as historical development from gesture to sign (Janzen, 2012, 2017):

Grammaticalization is the diachronic process by which lexical items develop into grammatical items in a language, or where items that are less grammatical in nature increase in their grammatical function (Heine et al., 1991; Bybee et al., 1994; Bybee, 2003; Hopper and Traugott, 2003; Brinton and Traugott, 2005; others). Grammaticalization in signed languages has been shown to develop by the same robust principles as for spoken languages with the exception that, whereas, for spoken language, historic sources for grammatical elements can only be shown to be earlier words, for signed language, grammatical elements can sometimes be traced back to gestural origins (Heine and Kuteva, 2007). Among such studies on ASL, Janzen (1998, 1999) has outlined the grammaticalization of topic marking as developing from a generalized questioning gesture, through regularized yes/no question marking, to topic marking. Janzen (1995) shows the development of the ASL lexical verb FINISH into both perfective and completive markers. Wilcox and Wilcox (1995), Shaffer (2000, 2002, 2004), Janzen and Shaffer (2002), and Shaffer and Janzen (2016) have outlined a number of ASL modals that have gestural sources for their development, and the evolution of discourse markers has been undertaken by Wilcox (1998)” (Janzen, 2017, p. 516–517).

Summing up, from a historical point of view, we observe gesture changes that are comparable to language change: *a historical dynamics of gesture and sign.* Gestural forms may stabilize (through repeated usages) and in some cases, undergo processes of lexicalization and grammaticalization and transform into signs within a signed language.

### Dynamic Relations Across Languages: Comparing Co-speech Gesture to Co-sign Gesture

Karen Emmorey’s provocative paper “Do signers gesture?*”* diagnosed a discontinuity assumption concerning the relation between co-speech gesture and co-sign gesture (Emmorey, 1999). In a recent discussion of this question, Janzen points out that although Emmorey discusses commonalities between co-speech and co-sign gestures, she concludes “that essentially a signer’s gestures are not like co-speech gestures” (Janzen, 2017, p. 514). Kendon (2004, p. 324) also underlines that Emmorey’s paper insinuates a sharp distinction between gesture and sign, while at the same time providing examples of “how signers may insert ‘gestures’ into their discourse” (Kendon, 2004, p. 324).

Liddell has argued that the ASL use of space, depicting verbs, pointing, and listing buoys is gestural (see Janzen, 2017, p. 515–518, and Kendon, 2004, p. 310–311 for discussions of this work). Janzen points out that while for Liddell “it is clear that he considers gestural material to exist pervasively in modern ASL” (Janzen, 2017, p. 516), nevertheless a sharp boundary between gesture and sign is established (Janzen, 2017, p. 518). Commonalities between co-speech and co-sign gestures have furthermore been discussed in the context of constructed or depicted action (Dudis, 2011; Janzen, 2017, p. 527; Hübl and Steinbach, 2018), classifier constructions (Supalla, 1982, 1986), or nominal proforms (Schembri, 2003; see Janzen, 2017, p. 525–526 and Kendon, 2004, p. 316–324 for detailed expositions).

Vermeerbergen and Demey (2007) suggest a simultaneity of gestures with spoken and signed language, and Goldin-Meadow and Brentari (2017, p. 1) “come to the conclusion that signers gesture just as speakers do.” Janzen proposes a usage-based, discourse-led approach to language as multimodal, as resting upon composite utterances: “Here we consider multimodality as a general characteristic of language, with composite utterances as instantiations of multimodality” (Janzen, 2017, p. 519). Following Enfield’s model, Janzen (2017, p. 518) defines utterance as “a complete unit of social action which always has multiple components, which is always embedded in a sequential context…, and whose interpretation always draws on both conventional and non-conventional signs, joined indexically as wholes (Enfield, 2009, p. 223).”

These proposals mark an important shift toward a comparative perspective between two fully fledged languages that are both multimodal. They indicate a path toward deepening and systematizing existing comparisons. One possible starting point for a systematic comparison would be to start either from a gesture studies or a sign language studies understanding of gestures. Starting from a gesture studies point of view could involve an investigation of the full spectrum of gestural forms (singular, recurrent, and emblematic gesture) in spoken and signed language use. For instance, Müller (2004) and Bressem and Müller (2014a) have documented that the palm-up-open-hand (PUOH) is widely used as a pragmatic gesture by German speakers. Steinbach has shown that it is frequently used in German Sign Language (DGS).

Starting, on the other hand, from a sign language point of view involves investigating how, for example, ‘constructed action’ (Bressem et al., 2018; Hübl and Steinbach, 2018), ‘classifiers’ (Schembri, 2003; Kendon, 2004; Müller, 2009), or the use of ‘space,’ ‘depicting verbs,’ or ‘buoys’ (Liddell, 2003) are potentially realized in co-speech gestures.

**FIGURE 7:**
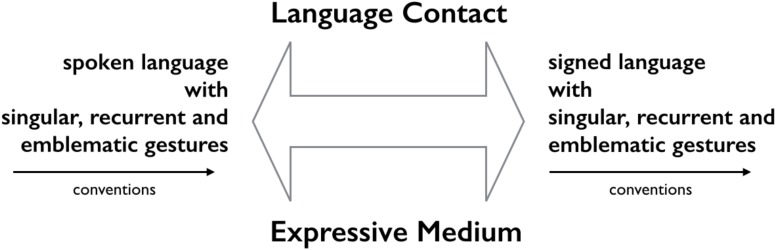
Comparing multimodal languages: media commonalities and language contact.

Such a comparison between two languages involves two facets: commonalities of gesture and sign resulting from a shared medium of expression (what Kendon refers to as being ‘cut from the same cloth’), and commonalities resulting from language use within and across language communities. In Germany, for instance, spoken German and German Sign Language are in close language contact (cf. **Figure 7**). This holds for the community of DGS signers (the use of the PUOH documents this), but it also affects bilingual speakers of German and DGS, who may include signs in their gesturing, much as they integrate a new Anglicism into their spoken language.

In sum, this points toward dynamic relations between co-speech gestures and co-sign gestures across signed and spoken languages. The dynamic relations are motivated either by the commonality of the medium of expression or by a language-contact situation.

## Conclusion

The systematic reconstruction of the gesture-sign relation across the history of gesture studies offered in this paper has argued that the question of how gesture and sign relate critically depends on the notion of ‘gesture’ employed. In fact, there is not one question at all, but rather a multitude of questions to be addressed. Minimally, one must separately address the question of gesture change, that is, the historical dynamics of gesture and sign, and the question of cross-linguistic comparison of spoken and signed languages. I have also shown that comparing multimodal languages in use, that involve singular, recurrent, and emblematic gestures, is different from comparing signing or speaking only with regard to singular gestures and under experimental conditions. Against this background, the ‘cataclysmic break’ diagnosed by Singleton et al. (1995) appears to be the result of the particular definition of the term ‘gesture,’ the experimental setting, and a static concept of language. Although restricting the term gesture to singular gestures makes sense in an experimental condition and to answer a specific psychologically motivated question (such as Goldin-Meadow’s focus on gestures that predict learning), it does, however, not tell us anything about other forms of gestures that we observe in speaking as well as in signing people. In fact, it excludes a broad range of gestural forms pre-hoc and thus hides that many gestures are partially or fully conventional and yet used with speech. It also makes it impossible to see that gestures differ in terms of conventionality and stabilization only gradually and not categorically. Moreover, experimental evidence based on this restrictive notion of gesture, such as the gestures speakers produce when forced to suppress speech (so-called ‘silent gesture’), is not adequate for countering linguistic observations concerning lexicalization processes that describe gesture change across time. Historical linguistics typically reconstructs processes of language change without recourse to psychological experiments. And, indeed, there is mounting evidence that, historically, not only certain lexical signs but also some grammatical ones have evolved from gesture (Wilcox and Wilcox, 1995; Janzen, 1998, 1999; Shaffer, 2000, 2002, 2004; Janzen and Shaffer, 2002; Wilcox, 2005, 2007, 2013; Wilcox et al., 2010; Shaffer and Janzen, 2016). In contrast to Goldin-Meadow and Brentari, and also in contrast to McNeill, this suggests a dynamic, continuous and ongoing process of historical change, where no cataclysmic break is involved, and no sudden rupture transforms gesture into sign from one moment to another.

Gestures produced under experimental conditions of suppressed speech cannot tell us whether speakers engaged in other discourses than narratives of visual stimuli produce gestural sequences that have more in common with ‘silent gestures’ than it appears. In fact, not much is known about gesture sequences, gesture scenarios, or local conventionalization processes occurring in naturalistic communicative contexts, but what little we know suggests that gestures are indeed often produced in complex structures involving linear as well as simultaneous productions of gestures (Müller, 2010; Müller and Tag, 2010; Müller et al., 2013a,b; Ladewig, 2014a; Bressem et al., 2018; Ladewig, in press). A narrow focus, useful for experiments, hides the full range of gestural forms commonly employed with spoken and signed language in naturalistic contexts. From a point of view of language use, this appears as a deliberate exclusion of the scope of phenomena that fall under a ‘composite utterance’ model. If we agree, however, that language is inherently (or ‘variably,’ Cienki, 2012) multimodal, then we need cross-linguistic investigations of spoken and signed languages along the lines set out in this paper. It suggests dynamic relations across multimodal languages that are motivated by commonalities of the expressive medium and by language contact.

This brings us back to the outset of this paper and to the milestone work carried out by Adam Kendon, David McNeill, and Susan Goldin-Meadow making it unmistakably clear that the study of gestures belongs to the study of language. The controversial positions concerning the relation between gesture and sign reflect different concepts of gesture and of language. From the point of view of studying multimodal language use ‘in the wild,’ as advocated in this article, the relation between gesture and sign is to be seen as dynamic on various levels, which in turn opens up fascinating new avenues for research.

## Author Contributions

The author confirms being the sole contributor of this work and approved it for publication.

## Conflict of Interest Statement

The author declares that the research was conducted in the absence of any commercial or financial relationships that could be construed as a potential conflict of interest.

## Footnotes

1 Note that although the author favors the use of the term ‘gesture’ over ‘visible action as utterance,’ the position advocated stands in the tradition of Kendon’s work on the relation between gesture and sign, and was initiated and inspired by his work early on.
